# Hemostatic Dysfunction in Dogs Naturally Infected with *Angiostrongylus vasorum*—A Narrative Review

**DOI:** 10.3390/pathogens11020249

**Published:** 2022-02-14

**Authors:** Jakob L. Willesen, Rebecca Langhorn, Lise N. Nielsen

**Affiliations:** Department of Veterinary Clinical Sciences, Faculty of Health and Medical Sciences, University of Copenhagen, Dyrlaegevej 16, 1870 Frederiksberg, Denmark; rel@sund.ku.dk (R.L.); lini@sund.ku.dk (L.N.N.)

**Keywords:** canine angiostrongylosis, French heartworm, bleeding, hemostasis, coagulopathy

## Abstract

This narrative review aims to describe *Angiostrongylus vasorum*-induced hemostatic dysfunction of dogs with emphasis on clinical and laboratory findings as well as potential therapeutic strategies for the bleeding patient. Canine angiostrongylosis (CA) is a disease with potentially high morbidity and mortality in endemic areas and with fatal outcome often associated with either severe respiratory compromise, pulmonary hypertension and right-sided heart failure, or hemostatic dysfunction with severe bleeding. The most common signs of hemorrhage are hematomas, petecchiation, ecchymoses, oral mucosal membrane bleeding and scleral bleeding, while intracranial and pulmonary hemorrhage are among the most severe. The pathophysiological mechanisms underlying hemostatic dysfunction in these patients are presently researched. While the larval effect on platelets remains unknown, the parasite appears to induce dysregulation of hemostatic proteins, with studies suggesting a mixture of pro-coagulant protein consumption and hyperfibrinolysis. Importantly, not all dogs display the same hemostatic abnormalities. Consequently, characterizing the hemostatic state of the individual patient is necessary, but has proven difficult with traditional coagulation tests. Global viscoelastic testing shows promise, but has limited availability in general practice. Treatment of *A. vasorum*-infected dogs with hemostatic dysfunction relies on anthelmintic treatment as well as therapy directed at the individual dog’s specific hemostatic alterations.

## 1. Introduction

Canine angiostrongylosis (CA) is a disease with potentially high morbidity and mortality in endemic areas. The disease is reported worldwide, currently with endemic occurrence throughout Europe (including United Kingdom), Eastern Canada, South America, and Uganda [1]. Recent years have shown an increase in the number of reported cases along with an expansion into new geographical areas [2,3,4]. The majority of the clinical cases are reported from European countries with sporadic cases reported from other parts of the world [5,6]. Canine angiostrongylosis is caused by the metastrongyloid nematode *Angiostrongylus vasorum* (*A. vasorum*) (Baillet, 1866), also known as the “French heartworm” [7]. The adult worms reside in the pulmonary arteries and the right side of the heart. Here eggs are deposited, whereafter L1 larvae are hatched and migrate through the pulmonary parenchyma before being coughed up, swallowed, and excreted with feces. The larvae molt from L1 into L2 and finally the infective L3 stage in snails and slugs. Dogs become infected when eating snails harboring the L3 stage, following which the larvae penetrate the gastric wall and enter the lymphatic system. The lymph carries them into the pulmonary arteries where development into adult worms takes place [5]. In a clinical setting, the diagnosis is most commonly reached either by demonstration of L1 larvae in fecal samples by use of the Baermann sedimentation technique or by demonstration of circulating parasitic antigen using a commercially available in-house test with good sensitivity and high specificity [8,9,10,11,12].

Migration of the L1 larvae through the pulmonary parenchyma and the resultant inflammation are thought to be responsible for the majority of clinical signs in infected dogs. The most common signs pertain to the respiratory tract, but, in some cases, dogs develop hemostatic dysfunction resulting in a variety of bleeding manifestations. Whereas survival for the mildly infected dog following treatment is almost 100% [13,14,15], a high proportion of dogs with hemostatic dysfunction may have a fatal outcome [16,17,18]. The underlying cause of bleeding in these patients is not fully clarified, but it appears that the hemostatic system may be affected at multiple different sites, even in the same animal [18,19]. This information is important, not only for understanding of disease pathophysiology, but even more so in order to tailor therapy to the needs of the individual patient, thereby improving prognosis.

This narrative review aims to describe the *A. vasorum*-induced hemostatic dysfunction of dogs with regard to clinical and laboratory findings, diagnostic possibilities, as well as potential therapeutic strategies. 

### Search String and Limitations

An open search was performed using Medline, Embase and CAB abstract archives using the terms “*Angiostrongylus vasorum,”* “*angiostrongylosis*,” “*bleeding*,” “*hemorrhage*,” “*hemostasis*,” “*coagulation*,” and “*coagulopathy*” from 1900 to present. All three authors performed the electronic search and reviewed the bibliographies of retrieved articles to identify further articles of interest. The authors then performed additional searches in relation to specific subtopics in the review. All peer-reviewed articles were considered for the review, including single case reports and case series.

## 2. Clinical Presentations of *Angiostrongylus vasorum*-Infected Dogs

The most frequently reported clinical signs in dogs diagnosed with *A. vasorum* infection are related to the respiratory system. Depending on the population described, cardiorespiratory signs such as cough, dyspnea, and exercise intolerance occur in around 40–65% of cases [16,17,18,20]. However, the absence of respiratory signs does not exclude *A. vasorum* infection, regardless of the severity of clinical signs. Some cases may even present with nonspecific signs only, including mild gastrointestinal signs. Infection with a fatal outcome is often associated with either severe respiratory compromise, pulmonary hypertension and right-sided heart failure, and/or hemostatic dysfunction with fatal bleeding [21,22,23,24]. 

**Figure 1:**
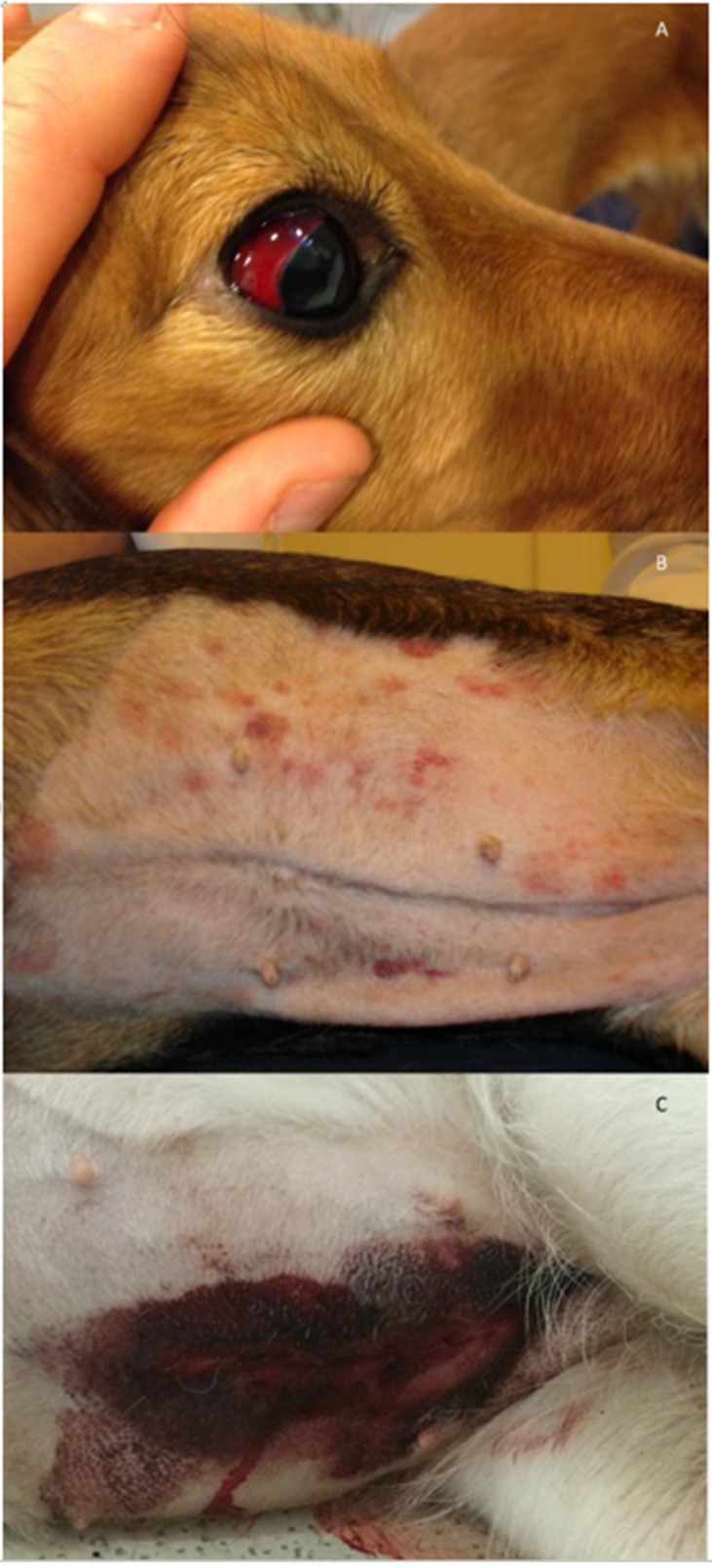
Examples of clinical presentations in dogs with hemostatic dysfunction due to *Angiostrongylus vasorum* infection. (**A**) Scleral bleeding in a ten-month-old Golden Retriever. (**B**) Petecchia and ecchymoses on the ventral abdomen of a four-year-old mixed breed dog. (**C**) Spontaneous wound bleeding after ovariectomy in a one-year-old German Shepherd. Photos by J.L.Willesen.

**Figure 2:**
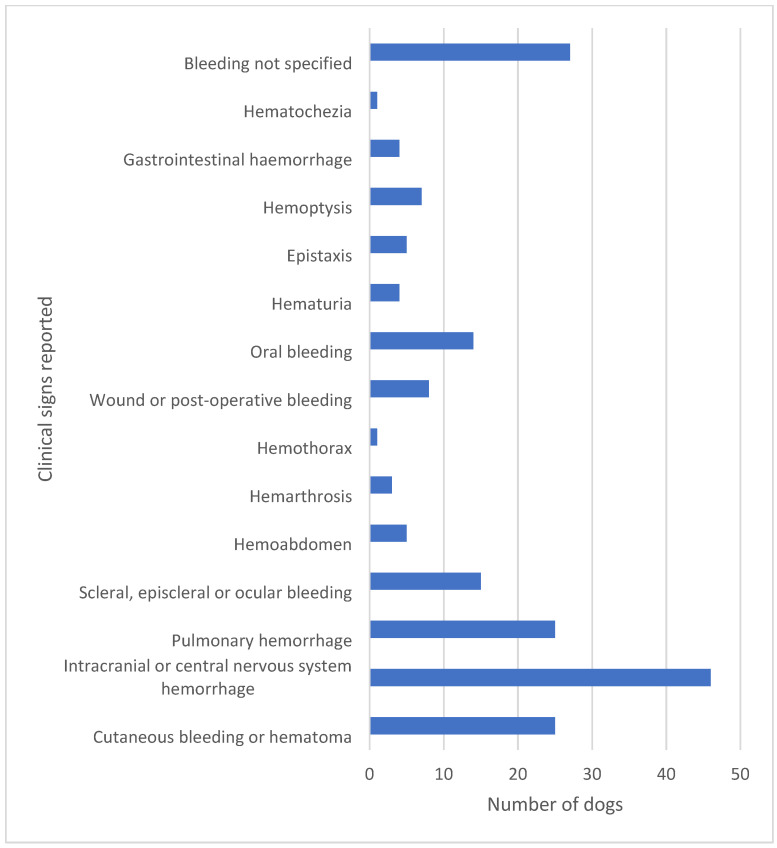
The clinical signs of bleeding reported in 143 cases in the literature are highly diverse. In a high proportion of dogs, more than one clinical sign of bleeding was reported. A fatal outcome was reported in 40 of these 143 dogs corresponding to 28% [15,16,17,18,22,23,26,27,28,29,30,31,32,33,34,35,36,37,38,39,40,41,42,43,44,45,46,47,48,49,50,51,52,53,54,55,56].

Dogs with hemostatic dysfunction secondary to CA present with a plethora of clinical signs. As the hemostatic dysfunction is often systemic rather than localized to the respiratory system, more than one clinical sign of bleeding may be present. In the literature, reported cases reflect the severity of clinical signs as well as the ability to detect bleeding by clinical examination and with the help of laboratory tests of hemostasis and diagnostic imaging. It is the authors’ opinion that, depending on location and visibility, minor bleeding may go unrecognized and thus may be underreported in the literature. Severe clinical cases are also more likely to be reported, especially when the disease is encountered in new geographical areas unfamiliar with its presentation [25,26,27]. The most common signs of hemorrhage are hematomas, petecchiation, ecchymoses, oral mucosal membrane bleeding, scleral bleeding, epistaxis, pulmonary hemorrhage, and wound bleeding following trauma or surgery [17,18] (Figure 1). However, rare presentations such as hemothorax, hemoabdomen, and hematuria have also been reported [23,28,29]. Intracranial and spinal cord hemorrhage are extensively reported due to an often fatal outcome [18,23,30] and are of particular concern along with severe pulmonary hemorrhage or major bleeding into body cavities. It is this variety of bleeding diathesis manifestations that can make the correct diagnosis difficult to reach if based solely on clinical signs. In hyperendemic areas, unexplained hemostatic dysfunction in any dog should give rise to suspicion of *A. vasorum* infection. When reviewing the literature, as many as 28% (40/143) of dogs presenting with bleeding diathesis will have a fatal outcome [15,16,17,18,22,23,26,27,28,29,30,31,32,33,34,35,36,37,38,39,40,41,42,43,44,45,46,47,48,49,50,51,52,53,54,55,56] (Figure 2). These numbers do, however, include some bias, as single cases with fatal outcomes may be more likely to be reported when encountered in new geographical areas. Despite this, even in referral practice settings in areas with endemic angiostrongylosis, a fatal outcome among *A. vasorum*-positive dogs with clinical bleeding is reported in the range 12.5–33.3% [18,31,46].

## 3. Physiology of Hemostasis

Hemostasis relies on interactions between endothelium, platelets, coagulation factors, fibrinolytic factors, their cofactors and inhibitors, as well as blood flow and vascular tone. Hemostasis is initiated by exposure of tissue factor (TF) through vessel wall injury, triggering a multitude of cellular and plasma protein interactions that culminate in clot formation [57,58]. The interaction between vessel wall and platelets, mediated by von Willebrand factor (vWF), was traditionally named primary hemostasis, while secondary hemostasis referred to a cascade of consecutively activated coagulation factors, resulting in the final fibrin clot. However, while this differentiation retains some applicability for teaching purposes, one must study the complexity of the cell-based model of hemostasis for comprehension of the mechanisms involved in hemostatic pathophysiology. 

Rather than being a singular cascade of events, hemostasis is explained by the cell-based model as overlapping stages known as initiation, amplification, and propagation [57,58]. Initiation takes place on exposed TF-bearing cells on which TF complexes with circulating coagulation factor (F) VII (which is then activated to FVIIa). The TF/FVIIa complex activates FIX and FX. Factor Xa activates and complexes with circulating FVa on the cell surface, culminating in activation of thrombin, however in too low a concentration for thrombin to induce fibrinogen cleavage. Thrombin must be activated on the platelet itself and in larger amounts for ultimate fibrin formation to take place. In the amplification stage, the hemostatic process therefore shifts to the surface of the platelet, which is activated by thrombin once attached to the site of vessel injury. Bound to the platelet, thrombin activates FV (released from platelet α granules), FVIII (cleaved from circulating complexes with vWF), and FXI (platelet-bound), preparing the platelet for pro-coagulant complex assembly. Factor IXa diffuses unhindered from the TF-bearing cell to the platelet, but additional FIX activation also takes place directly on the platelet surface by FXIa. With the formation of the FVIIIa/FIXa (tenase) complex on the platelet, the propagation stage commences, leading to activation of FX. The subsequent FXa/FVa (prothrombinase) complex assembly finally causes the thrombin burst necessary for both fibrinogen cleavage to fibrin and activation of FXIII, which cross-links and stabilizes the fibrin clot [57,58].

In order to prevent both bleeding and thrombosis, clot formation must be balanced by clot breakdown, i.e., fibrinolysis. Fibrinolysis was traditionally referred to as tertiary hemostasis because it will naturally follow the process of clot formation. In response to endothelial injury and to thrombin generation, tissue plasminogen activator (tPA) is released from the endothelium and binds to fibrin lysine residues in the clot. Only when bound in the actual clot can it convert plasminogen to plasmin, which ultimately lyses fibrin to D-dimers and other fibrinogen degradation products (FDPs) [43,58,59].

The processes of coagulation and fibrinolysis are counteracted by their inhibiting anticoagulant and antifibrinolytic systems, with important regulatory proteins including thrombomodulin, protein C and S, and antithrombin (anticoagulant) and plasminogen activator inhibitor 1 and thrombin-activatable fibrinolysis inhibitor (antifibrinolytic) [58]. 

## 4. Hemostatic Alterations in *A. vasorum* Infection

**Figure 3:**
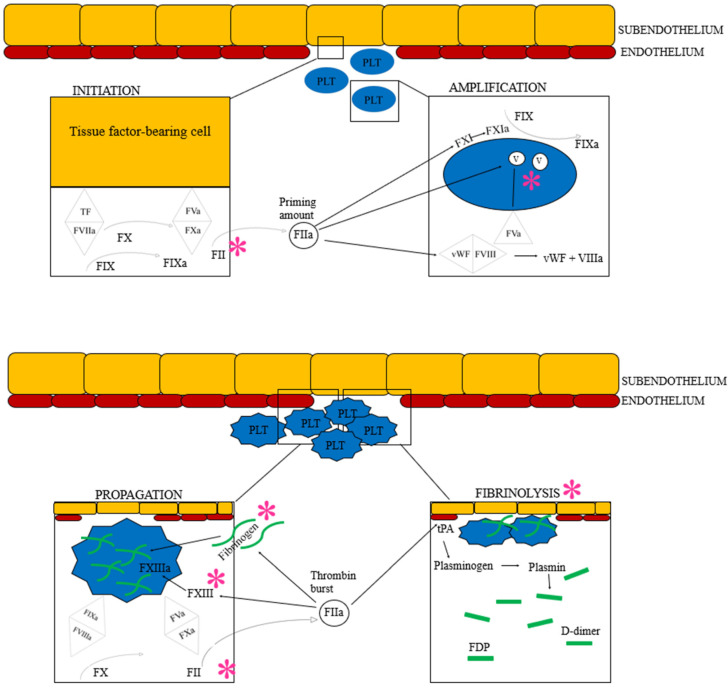
The cell-based model of hemostasis followed by fibrinolysis. Based on current understanding of the hemostatic pathophysiology of *Angiostrongylus vasorum* infection, each * represents the suspected factors or areas of hemostasis impacted by the parasite or its metabolic products: Coagulation factor V, II, I and XIII are believed to be suppressed directly or indirectly. Additionally, angiostrongylosis can cause primary hyperfibrinolysis. See text for details. F: Coagulation factor (the subsequent roman numeral is followed by an “a” when the factor is activated), FDP: Fibrinogen degradation product, PLT: Platelet, TF: Tissue factor, tPA: Tissue plasminogen activator, vWF: von Willebrand factor. Image created by the authors J.L. Willesen, R. Langhorn and L.N. Nielsen.

Several experimental set-ups, clinical case reports, and retrospective studies have reflected upon the exact pathophysiological mechanisms behind the noted hemostatic dysfunction in dogs with *A. vasorum* infection (Figure 3). However, these studies are difficult to compare. While experimental set-ups generally do not appear to result in overt clinical bleeding [60,61,62], common for most clinical studies of dogs with bleeding diathesis is the retrospective nature of the studies, variability of clinical presentations, and often less stringent laboratory investigations. 

Initial experimental studies in dogs suggested that *A. vasorum* infection caused an immune complex deposition and complement fixation systemically, leading to an inappropriate triggering and initiation of hemostasis. This would affect both platelets and coagulation factors and was believed to result in disseminated intravascular coagulation (DIC) in either a low grade or chronic form [61,63]. Case reports and retrospective clinical studies in dogs spontaneously infected by *A. vasorum* have similarly suggested a hypothesis of consumption of both platelets and coagulation factors [16,31,51]. However not all studies support the theory of DIC [64]. A recent sequential serum proteome study in eight dogs experimentally infected with *A. vasorum* revealed that several proteins important for physiological hemostasis were downregulated in the chronic phases of infection [64]. Among others, the proteins mannan-binding lectin serine peptidase 1 and 2 (MASP1, MASP2), coagulation factor V, coagulation factor XIII subunit b, a disintegrin and metallopeptidase with thrombospondin type 1 motif 13 (ADAMTS13), and histidine-rich glycoprotein (HRG) were shown to be of interest. The proteins MASP-1 and MASP-2 are associated with cleavage of both prothrombin, fibrinogen, factor XIII and thrombin-activatable factor inhibitor (TAFI), and their downregulation could reduce the formation of a stable clot. Specifically, the downregulation of factor V would affect the formation of the tenase complex necessary for thrombin burst generation in the propagation phase, and a reduced concentration of factor XIII subunit b could decrease or decelerate fibrin cross-linking. These findings suggest the creation of a less stable clot. Furthermore, a less stable clot combined with downregulation of the fibrinolysis inhibitor TAFI might also make the clot more prone to fibrinolysis. Not all findings of the study could explain an increased bleeding diathesis, however. A reduction in the enzyme ADAMTS13 would reduce vWf cleavage, leading to a firmer initial clot, while a lower concentration of HRG could both increase or decrease fibrinolysis [64]. 

A similar experimental sequential proteome study in foxes has also detected alteration in several hemostatic proteins leading to a combination of activation and suppression of specific hemostatic components. There were several distinctions between this fox study and the canine study; most essentially, however, it appears that foxes show stronger resistance to the infection [65].

Recent studies have found that hypofibrinogenemia is common in bleeding dogs with angiostrongylosis [19,31]. As an acute-phase reactant, fibrinogen would be expected to be increased in infection, and hypofibrinogenemia therefore is a conundrum in this population. An older study detected fibrinogen deposits in the pulmonary vessels themselves, suggesting that fibrinogen consumption was caused by intravascular coagulation, such as seen with DIC [63]. However, more recent studies suggest consumption through a different pathway, namely that of hyperfibrinolysis [19]. There have been a few case reports and more recently larger studies revealing hyperfibrinolysis in dogs with angiostrongylosis, almost exclusively in those presenting with hemorrhage [19,33,52]. Hypofibrinogenemia has been found significantly associated with hyperfibrinolysis in one study [19], but the mechanism behind the hyperfibrinolysis itself remains to be elucidated. One likely theory is direct endothelial injury caused by the parasite or its metabolic products [19]. Endothelial injury is known to be an initiator of hyperfibrinolysis due to resultant release of tPA [66,67]. Another possibility is specific interaction of the parasite with the fibrinolytic system, a mechanism which has been reported for *Dirofilaria immitis.* Antigens from *D. immitis* have been shown experimentally to enhance effects of plasminogen activators and also to directly bind to plasminogen, thereby themselves inducing the conversion to plasmin [68,69,70]. Finally, while hyperfibrinolysis might cause hypofibrinogenemia through consumption, patients might also be more prone to hyperfibrinolysis secondary to formation of a less stable clot (due to hypofibrinogenemia among other factors) as explained above. Hence, a co-dependency of hypofibrinogenemia and hyperfibrinolysis is likely. 

Acquired von Willebrand syndrome has been detected in a few case reports of dogs with *A. vasorum* infection [39,55]. The dogs presented with a reduced percentage of vWf antigen (vWf:ag), either alone or in combination with a reduced concentration of coagulation factor VIII. In both cases, the vWf:ag test normalized after commencing antiparasitic therapy. Acquired von Willebrand syndrome is a well-known syndrome in people, characterized by clinical bleeding and laboratory findings compatible with inherited von Willebrand disease, but without a personal or family history of bleeding diathesis. Von Willebrand syndrome may occur in relation to a number of diseases causing either a reduced production of vWf, an increased breakdown, or an increased binding of vWf to either platelets or to autoantibodies disturbing the initial platelet clot formation [71]. The underlying causes of transient low percentages of vWf in the two case reports of dogs were not further explored. 

Thrombocytopenia is common in dogs with angiostrongylosis, but is generally only mild-moderate [16,51,72], i.e., in a range not itself sufficient to cause clinical bleeding. Its cause could potentially be platelet consumption secondary to other hemostatic abnormalities. Secondary immune-mediated thrombocytopenia has been suggested in two case reports of dogs with angistrongylosis and severe thrombocytopenia [38,42], but only confirmed by anti-platelet antibody testing in one [38]. Finally, it has been speculated that an acquired thrombocytopathy may be present in dogs with *A. vasorum* [31,73], but evidence for this is lacking.

While most of the above findings have led to clinical bleeding, some patients in fact present hypercoagulable [31,74]. Indeed a recent study showed that *A. vasorum* has the ability to induce neutrofil extracellular entrapment [75], a finding frequently associated with hypercoagulability [76].

## 5. Laboratory Findings and Assessment of Hemostasis Testing

Studies have examined the described clinical hemostatic findings by use of several different laboratory analyses in an attempt to localize an anomaly in either initiation, propagation/amplification or fibrinolysis — or a combination of the above.

### 5.1. Platelets and von Willebrand Factor

Platelet count differs significantly in dogs with *A. vasorum* infection. While some patients present with a platelet count within reference range, they more commonly present with mild to moderate thrombocytopenia [16,51,72]. Severe thrombocytopenia with a platelet count below 50 × 10^9^/L is rare [38]. 

Specific analyses for a thrombocytopathy would include different platelet function tests analyzing for platelet adhesion, secretion, and aggregometry [77,78]. The literature search carried out for this publication failed to identify studies that have examined platelet function in dogs with angiostrongylosis.

Apart from the two case reports suggesting acquired von Willebrand syndrome [39,55], one larger retrospective study did not identify any difference in vWf antigen (vWf:ag) between bleeding and non-bleeding dogs with angiostrongylosis [31]. In human medicine, acquired von Willebrand syndrome is diagnosed by examining a combination of vWf:ag, vWf activity assays, collagen binding assays, and multimer analysis [79], tests which are not readily available in canine medicine.

### 5.2. Coagulation Factor Analyses

The most common hemostatic tests applied in dogs with angiostrongylosis in the literature are activated partial thromboplastin time (aPTT) and prothrombin time (PT). Patients have presented with bleeding diathesis with either of or both aPTT and PT prolonged [16,22,23,29,31,35,43,51,80]. In one of the largest retrospective studies comparing *A. vasorum*-positive dogs with and without bleeding diathesis, a significantly larger proportion of bleeding dogs had prolonged aPTT or PT compared to non-bleeding dogs [16]. However, several case reports and case series have also found both parameters within normal limits in *A. vasorum*-positive dogs with bleeding diathesis [23,30,36,38,39,55,56], and one larger study failed to show a significant difference in PT and aPTT between dogs with and without bleeding diathesis [31]. The equivocal results of these tests could be related to the wide range of different analyses offered commercially, which include different concentrations and compositions. The tests can be performed on either citrate-stabilized whole blood or plasma, can be analyzed in smaller table-size instruments as well as in larger reference laboratories, and, within the test set-up, can be initiated by a range of different activating agents, which ultimately will affect the analytical sensitivity and specificity for detection of bleeding [74,81,82,83]. However, it is also possible that coagulation factors are unaffected in some bleeding dogs with angiostrongylosis. 

A few studies have expanded their routine hemostasis laboratory test panels in their efforts to characterize the hemostatic dysfunction seen in dogs with *A. vasorum* infection. Single coagulation factors have been examined in a case report identifying a reduced FVIII and FIX in a bleeding dog [23], and both FV and FVIII were reduced in non-bleeding dogs compared to controls in 10 dogs experimentally infected with *A. vasorum* [84]. 

Fibrinogen concentration has only been measured in a smaller number of studies. While some dogs have fibrinogen concentrations within the reference range [51], fibrinogen is now increasingly reported to be below reference in bleeding dogs [19,28,43] or lower in *A vasorum*-positive dogs with bleeding diathesis compared to non-bleeding dogs [31]. Also examining for fibrinogen deficiency, a thrombin clotting test was prolonged in three dogs with neurological bleeding diathesis secondary to *A. vasorum* infection [22].

### 5.3. Fibrinolysis Testing

To assess *A. vasorum*-positive patients for fibrinolysis, a small number of studies have examined the more non-specific FDPs, which are markers of both degradation of fibrinogen and cross-linked fibrin, and D-dimer concentration, which is a more specific test for the breakdown of cross-linked fibrin. Two case reports and a small case series of four cases found normal FDPs in one dog and increased FDPs in three dogs [22,38,56]. Two case reports and a number of case series all identified an increased D-dimer concentration [29,30,35,43,56,80], while one of the largest studies examining dogs with angiostrongylosis did not identify a difference between D-dimer concentration in dogs with or without bleeding diathesis [31]. However, more detailed evaluation of fibrinolysis in patients with angiostrongylosis has become available with the use of global hemostatic tests. 

### 5.4. Global Hemostasis Testing—From Initiation of the Clot to Fibrinolysis in One Test

**Figure 4:**
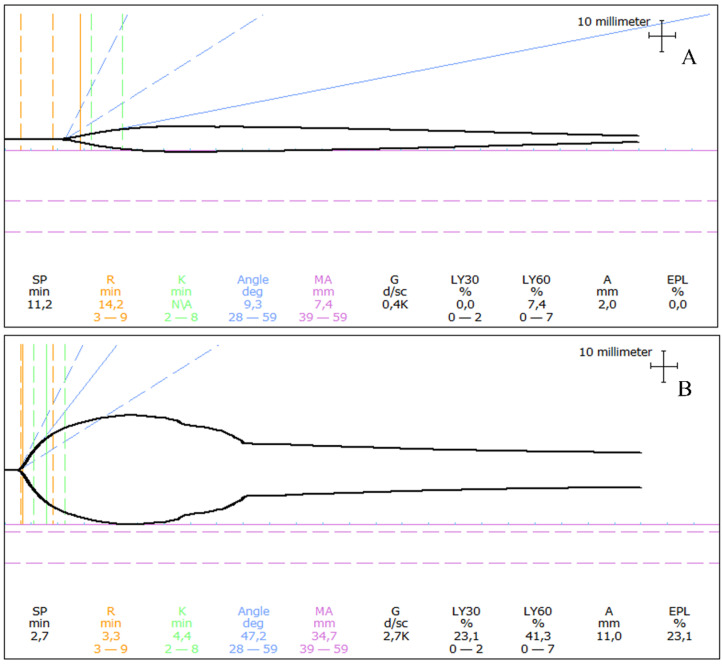
Examples of thromboelastograms in dogs with angiostrongylosis and clinical bleeding. (**A**) A one-year-old Labrador Retriever presented following a 30 s generalized seizure and with a history of prolonged bleeding from a paw pad wound a few weeks earlier. The thromboelastogram revealed prolongation of initiation (prolonged R) as well as severe hypocoagulability (severely decreased MA). (**B**) A one-year-old Samoyed presented with a small continuously bleeding wound on her tongue. The thromboelastogram revealed unremarkable initiation of coagulation (R within reference), mild hypocoagulability (mildly decreased MA), and severe hyperfibrinolysis (severely increased LY30 and LY60). R: Reaction time, MA: Maximum amplitude, LY30: Percentage of lysis 30 min following MA, LY60: Percentage of lysis 60 min following MA Thromboelastograms of *Angiostrongylus vasorum*-infected dogs seen by the authors at the University Hospital for Companion Animals, University of Copenhagen.

Viscoelastic testing, using either thromboelastography (TEG^®^) or rotational thromboelastometry (ROTEM^®^), has been applied in dogs with *A. vasorum* infection with interesting results (Figure 4). 

As these laboratory tests are performed in citrate-stabilized whole blood, the tests contain all the cellular and protein components required to mimic the hemostatic process in a more physiological environment, displaying the hemostatic process from initiation to clot formation and further into fibrinolysis (Table 1).

While the TEG^®^ is either initiated spontaneously or by using an agonist such as kaolin or diluted TF, the ROTEM^®^ assay includes several subtypes of tests with different agonists examining hemostasis from different aspects (Table 2).

A few case reports have shown hypocoagulable TEG^®^ parameters [33,43], while a larger study in *A. vasorum*-positive dogs with and without bleeding diathesis had more specific findings. In brief, Adamantos et al. (2015) analysed TEG^®^ in 20 *A. vasorum*-infected dogs and showed a prolonged R, low angle, low MA and G in dogs with bleeding diathesis (n = 18) compared to dogs without bleeding (n = 2) [31], while a single case report performed a modification to the TEG^®^ with the addition of tissue plasminogen activator (tPA) to improve the sensitivity for detection of hyperfibrinolysis and identified this in a dog with *A. vasorum* infection [33]. 

Using TEG^®^ parameters, a few studies have reported hypercoagulability in dogs with *A. vasorum* infection [31,74], none of which showed clinical bleeding. In our hospital using the same global hemostatic testing, the authors have, however, encountered rare hypercoagulable patients with concurrent bleeding diathesis, for which the cause of bleeding was not established using either of the described diagnostic tests. 

One research centre has focused on the use of ROTEM^®^ using ExTEM, InTEM, FibTEM, and ApTEM to examine bleeding diathesis in dogs with *A. vasorum* infection [19,52,72]. One retrospective study of 21 dogs revealed hyperfibrinolysis in 67%, most of these also hypofibrinogenemic [19]. In a more recent prospective study including 18 dogs with *A. vasorum* infection, specifically the use of CFT in ExTEM could indicate if dogs had bleeding diathesis, were hypocoagulable, or hyperfibrinolytic, compared to healthy dogs [52]. 

## 6. Therapeutic Strategies for the Bleeding Patient

The therapeutic strategy for the bleeding patient consists of two key elements — the anthelmintic treatment protocol and therapy directed specifically at the hemostatic alterations of the individual patient. 

### 6.1. Anthelmintic Therapy in the Bleeding Patient—Treatment of Choice

Prompt and correct anthelmintic treatment is the most important part of treating any dog with hemostatic dysfunction secondary to *A. vasorum* infection. Historically, several different treatment protocols have been reported using levamisole, ivermectin or fenbendazole in single cases or small case series [37,87]. More recently, novel anthelmintic therapeutics belonging to the group of macrocyclic lactones such as moxidectin and milbemycin oxime have been developed and investigated more systematically for use in canine angiostrongylosis [13,14]. Fenbendazole remains widely used as an off-label treatment [88], and the efficacy and safety of this drug in various dosages are comparable to treatment protocols using moxidectin and milbemycin oxime in naturally infected (as well as experimentally infected) dogs (Table 3) [13,16]. Currently, these three anthelmintics are used for treatment of most *A. vasorum*-infected dogs in most countries [88]. So far, fenbendazole is the most universally reported drug used in dogs with hemostatic dysfunction due to *A. vasorum* infections [88]. The use of moxidectin/imidacloprid spot-on solution has also been reported in dogs with hemostatic dysfunction, although only in very few cases [15]. A more systematic investigation of the use of different treatment protocols in this subgroup of dogs is therefore still absent. 

### 6.2. Prophylactic Therapy

For dogs recovering from *A. vasorum* infection with concurrent hemostatic dysfunction, the use of prophylactic treatment is recommended by the authors following successful treatment of the infection to prevent immediate re-infection. The spot-on formulation of imidacloprid/moxidectin, milbemycin oxime or moxidectin as tablets have all been approved for prevention of *A. vasorum* infection [13,14,89,90,91,92,93,94,95] (Table 3). Besides these cases, prophylaxis may also be recommended in dogs with a particular high risk of infection (e.g., dogs living in highly endemic areas, dogs with a high-risk behavior such as scavenging, coprophagia, or slug/snail ingestion, dogs undergoing planned surgery, or dogs younger than 1½ years of age, as dogs this young are commonly infected) [5].

**Table 3:** Published anthelmintic protocols for the treatment and prophylaxis of *Angiostrongylus vasorum* infections in dogs.

Therapeutic Protocols	Dosage	Study Design	Number of Dogs	Reference
Moxidectin/imidacloprid	0.1 mL/kg spot-on 10% moxidectin/2.5% imidacloprid. Single dose	Controlled, randomized, blinded, multicenter field trial study	23	[13]
Milbemycin	0.5 mg/kg PO once weekly for 4 weeks	Retrospective study of naturally infected dogs	16	[14]
Fenbendazole	25 mg/kg PO, SID for 20 days	Controlled, randomized, blinded, multicenter field trial study	27	[13]
Fenbendazole	50 mg/kg PO, SID for 5-21 days	Retrospective study of naturally infected dogs	23	[16]
**Prophylactic protocols**				
Moxidectin/imidacloprid	0.1 mL/kg spot on of 10% moxidectin/2.5% imidacloprid	Controlled, randomized, blinded dose confirmation study	24	[94]
Milbemycin oxime	0.5 mg/kg PO once weekly for 4 weeks	Placebo-controlled, randomized experimental study	40	[95]
Moxidectin/sarolaner/pyrantel	24 µg/kg moxidectin/1.2 mg/kg sarolaner/5 mg/kg pyrantel PO. Single dose	Placebo-controlled, blinded, randomized laboratory studies	32	[89]
Moxidectin/sarolaner/pyrantel	24 µg/kg moxidectin/1.2 mg/kg sarolaner/5 mg/kg pyrantel PO. Monthly dose	Randomized, placebo controlled, double-blinded, multicenter field trial study	622	[90]
Moxidectin/imidacloprid	0.1 mL/kg spot on of 10% moxidectin/2.5% imidacloprid	Controlled, randomized, and blinded experimental study	24	[91]
Spinosad/milbemycin oxime	45–60 mg/kg spinosad/0.75-1.0 mg/kg milbemycin oxime PO	Controlled, randomized, partly blinded laboratory study	16	[92]
Milbemycin oxime/afoxolaner	0.5 mg/kg milbemycin oxime/2.5 mg/kg afoxolaner PO. Monthly dose	Controlled experimental study	20	[93]

### 6.3. Targeting Individual Hemostatic Abnormalities

Given the multiple pathways through which *A. vasorum* may alter hemostasis, it follows that therapy for dogs presenting with clinical bleeding must be tailored to the individual based on the specific hemostatic abnormalities it exhibits. Typically, the hemostatic phenotypes that require specific treatment involve either coagulation factor deficiencies, primary hyperfibrinolysis (with or without hypofibrinogenemia) or a combination of these.

For dogs with coagulation factor deficiencies, the immediate requirement is replenishment of these factors. Fresh frozen plasma (FFP) is the treatment of choice [5,31], and it is the experience of the authors that a single transfusion of 10 mL/kg will generally suffice. TEG^®^ parameters can be used as a treatment guide, confirming the need and optimizing the dose for the individual dog [43].

For bleeding patients exhibiting primary hyperfibrinolysis, antifibrinolytic therapy is indicated. It is important to establish that the patient is normo- or hypocoagulable before initiating such therapy in order to confirm that hyperfibrinolysis is of the primary form. Both TEG^®^ and ROTEM^®^ analysis may assist in this evaluation. A hypercoagulable tracing with hyperfibrinolysis would suggest that hyperfibrinolysis is an appropriate response (so-called secondary hyperfibrinolysis, occurring secondary to a prothrombotic state) and should not be treated [96]. Secondary hyperfibrinolysis has not been reported in canine angiostrongylosis, however. The treatment regimen for primary hyperfibrinolysis has yet to be established with certainty, but tranexamic acid has been reported as the chosen treatment in several studies with published dosages ranging from 10–20 mg/kg administered slowly iv three times daily (TID) until hemorrhage resolves [19,33,59]. Higher dosages are likely to induce nausea [97,98], and the authors generally apply a dosage of 10 mg/kg iv TID. As the drug is primarily renally excreted, dose reduction is recommended in patients with renal impairment [59]. Alternative antifibrinolytic drugs include epsilon-aminocaproic acid and aprotinin, but their use has not yet been reported for this patient population. Length of therapy is similarly debated. While a single therapeutic intervention is generally sufficient for patients with factor deficiency-induced bleeding, it appears that hyperfibrinolysis is more persistent. In the experience of the authors, it is common for a follow-up TEG^®^ analysis to return to a hyperfibrinolytic tracing after cessation of antifibrinolytic therapy, signaling a risk for renewed bleeding. Accordingly, once hemorrhage has ceased, and intravenous therapy has been discontinued, the authors recommend oral tranexamic acid therapy (10 mg/kg po BID-TID) for at least three additional days.

For patients that develop hypofibrinogenemia along with hyperfibrinolysis, a hypocoagulable state will commonly occur as well, and antifibrinolytic therapy may not be sufficient to resolve the bleeding. In these cases, replenishment of fibrinogen is recommended concurrently. Hypofibrinogenemia in angiostrongylus patients often requires higher dosages of FFP than for other coagulation factor deficiencies [19], likely because treatment of hyperfibrinolysis is required concurrently in order to stop the consumption. Cryoprecipitate is an alternative to FFP, allowing for a lesser colloid volume to be infused and for more specific therapy to be administered by delivering primarily the larger coagulation factors (such as fibrinogen) concentrated in such a product [99]. 

While thrombocytopenia is common in patients with canine angiostrongylosis, it rarely reaches a level requiring specific therapy [16,51,72] and can be expected to normalize with antiparasitic treatment itself as well as with correction of other hemostatic abnormalities that may contribute to platelet consumption. In the rare case that develops immune-mediated thrombocytopenia secondary to angiostrongylosis [38], it is also of main concern to treat the underlying disease and any concurrent hemostatic abnormalities. However, if the patient bleeds due to thrombocytopenia alone (suspected in patients with platelet counts < 50 × 10^9^/L for which other hemostatic abnormalities are ruled out), a short course of immunosuppressive therapy with corticosteroids may be warranted [100]. It may be considered in critical cases to administer either a platelet transfusion (if platelet products are available) or a fresh whole blood transfusion in order to raise the platelet count (albeit only a minimal increase can be expected with fresh whole blood [43]) while awaiting the effect of immunosuppressives [101]. Alternatively, FFP or cryoprecipitate may be considered for supplying additional vWf, optimizing the interaction of the few available platelets with the endothelium. Similarly, desmopressin may be considered, as this increases the release of endothelial vWf, but these effects have only a very brief duration [102,103]. For patients with acquired von Willebrand syndrome secondary to angiostrongylosis, FFP/cryoprecipitate or desmopressin may be similarly considered, but, as for thrombocytopenia, this abnormality is highly unlikely to reach treatment-requiring levels [31]. One case report described an improvement of buccal mucosal bleeding time after desmopressin treatment in a patient with acquired von Willebrand syndrome secondary to angiostrongylosis [55].

For the bleeding patient that presents with or develops clinical signs of hypoxia due to anemia, it is recommended to also transfuse with erythrocytes, either as packed RBCs or whole blood.

## 7. Conclusions

*Angiostrongylus vasorum*-positive dogs with bleeding diathesis present with an extremely variable bleeding phenotype ranging from subtle hematomas or small ecchymoses to severe hemorrhage with fatal consequences. While routine coagulation tests such as aPTT and PT offer some information in these patients, newer global hemostasis tests provide insight into initiation, amplification, and propagation of coagulation as well as fibrinolysis, allowing for characterization of the specific hemostatic alterations of individual patients. This individual assessment of each *A. vasorum*-infected dog with clinical bleeding is important in order to tailor the treatment plan accordingly. At present, prognosis remains guarded in dogs presenting with hemostatic dysfunction due to angiostrongylosis, but improves with early recognition and tailored therapy. 

## 8. Future Perspectives for the Bleeding Patient with Angiostrongylosis

Studies have documented bleeding diathesis in dogs with angiostrongylosis since the 1980s. Although we have made several advances since then with regard to understanding of the pathophysiology, diagnostic tools available, and therapeutic regimens, several questions remain. Recent studies have identified downregulation of essential circulatory proteins affecting normal hemostasis, but how do these dysregulated proteins reflect local host-parasite crosstalk and interaction within the canine organism? How exactly does the parasite interact with these hemostatic proteins? Furthermore, why do some dogs with angiostrongylosis present with bleeding diathesis while others do not, and is the risk of bleeding related to specific host factors, parasitic burden, or chronicity of disease? Improved understanding of the hemostatic dysfunction is essential to guide and support future larger treatment studies and thereby clarify if current standards of therapy can be further optimized.

## Figures and Tables

**Table 1:** A selection of the most used parameters in TEG^®^ and ROTEM^®^ [85].

TEG^®^	ROTEM^®^	Definition	Clinical Utility
Reaction time/R (minutes)	Clotting time/CT (minutes)	Time to 2 mm amplitude	Measure of coagulation factors
Angle/α (degrees)	Angle (degrees)	TEG^®^ (slope between R and K) ROTEM^®^ (slope at tangent at 2 mm amplitude)	Measure of platelets, coagulation factors, and fibrinogen
Kinetics/K (minutes)	Clot formation time /CFT (minutes)	Time from 2 to 20 mm	Early indicator of clot kinetics. Measure of platelets, coagulation factors, and fibrinogen
Maximum amplitude/MA (mm)	Maximum clot firmness/MCF (mm)	Maximum amplitude	Measure of platelets, fibrinogen, and factor XIII
G (dyn/cm^3^)	-	Overall (global) clot strength calculated as (5000*MA)/(100-MA)	Measure of platelets, fibrinogen, and factor XIII
LY30 (%)	LI30 (%)	Lysis at 30 min after MA/MCF	Measure of fibrinolysis at 30 min after complete clot formation
LY60 (%)	LI60 (%)	Lysis at 60 min after MA/MCF	Measure of fibrinolysis at 60 min after complete clot formation
-	ML	Maximum Lysis	Maximum fibrinolysis during the analysis

**Table 2:** Selected ROTEM^®^ analyses performed in dogs with *A. vasorum* [86].

Test Name	Clinical Utility
ExTEM	Tissue factor activation, sensitive for factor deficiencies in the extrinsic system
InTEM	Contact factor activation, sensitive for factor deficiencies in the intrinsic system
ApTEM	Fibrinolysis inhibition by the addition of aprotinin
FibTEM	Determination of fibrinogen concentration
